# Lymph node metastasis in grossly apparent clinical stage Ia epithelial ovarian cancer: Hacettepe experience and review of literature

**DOI:** 10.1186/1477-7819-8-106

**Published:** 2010-11-30

**Authors:** Guldeniz Aksan Desteli, Murat Gultekin, Alp Usubutun, Kunter Yuce, Ali Ayhan

**Affiliations:** 1 Department of Obstetrics and Gynecology, Baskent University Faculty of Medicine, Ankara, Turkey; 2 Department of Obstetrics and Gynecology, Turkish Ministry of Health, Cancer Control Department, Ankara, Turkey; 3 Department of Pathology, Hacettepe University Faculty of Medicine, Ankara, Turkey; 4 Department of Obstetrics and Gynecology, Hacettepe University Faculty of Medicine, Ankara, Turkey

## Abstract

**Background:**

Lymphadenectomy is an integral part of the staging system of epithelial ovarian cancer. However, the extent of lymphadenectomy in the early stages of ovarian cancer is controversial. The objective of this study was to identify the lymph node involvement in unilateral epithelial ovarian cancer apparently confined to the one ovary (clinical stage Ia).

**Methods:**

A prospective study of clinical stage I ovarian cancer patients is presented. Patient's characteristics and tumor histopathology were the variables evaluated.

**Results:**

Thirty three ovarian cancer patients with intact ovarian capsule were evaluated. Intraoperatively, neither of the patients had surface involvement, adhesions, ascites or palpable lymph nodes (supposed to be clinical stage Ia). The mean age of the study group was 55.3 ± 11.8. All patients were surgically staged and have undergone a systematic pelvic and paraaortic lymphadenectomy. Final surgicopathologic reports revealed capsular involvement in seven patients (21.2%), contralateral ovarian involvement in two (6%) and omental metastasis in one (3%) patient. There were two patients (6%) with lymph node involvement. One of the two lymph node metastasis was solely in paraaortic node and the other metastasis was in ipsilateral pelvic lymph node. Ovarian capsule was intact in all of the patients with lymph node involvement and the tumor was grade 3.

**Conclusion:**

In clinical stage Ia ovarian cancer patients, there may be a risk of paraaortic and pelvic lymph node metastasis. Further studies with larger sample size are needed for an exact conclusion.

## Background

Epithelial ovarian carcinoma (EOC) is a lethal genital malignancy [1]. Only one third of cases are diagnosed in the early stages of the disease. Lymphadenectomy is an integral part of surgical staging and treatment for ovarian cancers, and they have a potential role in both staging and retroperitoneal debulking. Lymphatic node metastasis results in a change from stage I to stage IIIC. 5-year survival decreases from more than 90% to 20% to 60% if there lymphatic node metastasis is present and adjuvant therapy is needed [2-4]. However, there is debate on the extent of lymphadenectomy, particularly in early staged unilateral tumors (confined to only one ovary) [5,6]. Despite a detailed history of lymphadenectomies in scientific literature, there are only a limited number of reports analyzing this topic [5-7]. Furthermore, they are all of a retrospective nature and only include a small number of patients. The staging procedures of these studies and the extent of lymphadenectomies performed are also debatable. In this study, we aim to analyze the role and the extent of systematic lymphadenectomies and the lymphatic metastatic pattern of unilateral clinical stage Ia ovarian cancers in a prospective pattern, which is the first of its kind in available published literature. Since the number of such patients is low, the results were compared and evaluated with the results found in the literature.

## Methods

Two hundred and ninety-three consecutive patients (n = 293) were operated for primary epithelial ovarian carcinoma at Hacettepe University Faculty of Medicine, between January 2004 to April 2007. Fifty-seven (n = 57) of these patients (19.4%) were diagnosed with tumors confined to only one ovary based on both pre-operative and intra-operative evaluations. All the clinico-pathological variables of these patients are evaluated by using SPSS version 13.0.

The presence of the following factors were used as exclusion criteria for this study (clinical stage Ib and Ic are excluded):

a) Ruptured capsule (intraoperative or postoperative)

b) Presence of ascites

c) Presence of adhesions with neighboring tissues

d) Presence of gross or suspicious tumor on the surface of the capsule

e) Suspicious tumoral involvement of the contralateral ovary

f) Palpable lymphadenopathy suspicious for tumor metastasis

g) Presence of other concomitant cancers or cancers other than primary epithelial ovarian carcinoma

h) Patients incompletely staged at an external medical center or laparoscopy performed before admission

i) Patients undergoing fertility sparing surgery.

According to the inclusion criteria, twenty-four (n = 24) patients were excluded from the study and the remaining thirty-three (n = 33) patients were eligible for final analysis.

Patient age, gravida, parity and menopausal status, initial complaints, pre-operative Ca-125 levels, preoperative ultrasonographic appearence, intraoperative findings (adhesions, capsular involvement or rupture, tumor bilaterality, presence of ascites or lymphadenopaties or tumoral implants) and final pathological reports (maximal tumor size, presence of tumor on the contralateral ovary, capsular involvement, involvement of peritoneum or positive cytology, tumor histology, grade, clinical stage and the number of resected and metastatic lymph nodes) were the variables collected and analyzed prospectively in this study.

All patients were subjected to a primary surgical staging procedure by the same surgical team led by one of the authors and evaluated by the same pathologist, according to the FIGO recommendations. After a midline vertical incision, peritoneal washings were obtained from the pelvis, para-colic gutters, and diaphragm and then submitted for cytology. Multiple peritoneal biopsies from both suspicious and normal appearing areas were taken, together with the sampling of the diaphragmatic peritoneum if it was suspicious. All the peritoneal surfaces and solid organs were explored by inspection and palpation including the intestinal mesentery, and biopsies were taken from multiple sites. A total abdominal hysterectomy, bilateral salpingo-oopherectomy, and a total omentectomy were the initial steps of the surgery. After an adequate examination of the pelvis, a systematic pelvic and para-aortic lymphadenectomy was undertaken. A pelvic lymphadenectomy was accomplished by completely skeletonizing the external iliac vessels and removing all the nodes around the vessels. The common iliac and obturator nodes were dissected using blunt and sharp dissection, and all the tissues above the obturator nerve were removed. The para-aortic area was exposed just above the bifurcation. The retroperitoneal space and the lymph nodes at the bifurcation of the aorta anterior to the vena cava and below the renal vessels on both sides were dissected. Resected pelvic nodes were subdivided as right or left sided. However, para-aortic nodes could not be subdivided in a similar way due to the en bloc resection technique we used in the para-aortic region. The mean number of resected lymph nodes was 35 (range, 23-74), and the mean number of resected para-aortic lymph nodes was 10 (range 8-16). Finally, appendectomies were performed for all patients if not previously performed.

There were no severe complications attributable to the surgery. There was only one bladder injury. Three patients had postoperative morbidity: one had an intra-abdominal infection, one had a deep venous thrombo-embolism and the other patient had an intestinal obstruction that required another operation for a bridectomy. The overall morbidity rate was 12% (4/33). Following the final pathologic reports, patients with localized microscopic disease were accepted as upstaged. All patients except for stage Ia-Ib grade 1-2 disease received adjuvant combination chemotherapy (six cycles of paclitaxel plus carboplatin). Patients with unfavorable tumor histology (clear cell, transient cell or undifferentiated tumors) also received adjuvant chemotherapy, regardless of their stage or tumor grade.

The median follow-up of the patients was 16.1 months (range 1-37). The follow-up period was evaluated from the date of the operation to the date of the last follow up. One patient with clear cell histology developed a vaginal cuff recurrence shortly after the initial six cycles of chemotherapy. Recurrent disease was treated with salvage chemotherapy.

## Results

The median age of the patients was 55.3 (range 31-82). Eleven patients (33.3%) were <50 and twenty-two patients (66.6%) were ≥50. The preoperative Ca-125 level was ≤35 IU/L in 12 patients (36.4%), between 35-500 in 17 patients (51.5%) and ≥500 in the remaining four patients (12.1%). Tumor histology was serous in seven patients (22.1%), mucinous in eight patients (24.2%), endometrioid in five patients (15.2%) and clear cell in four patients (12.1%). The remaining nine patients (26.2%) had rare tumor histology's (mixed epithelial type in four, transitional in three, anaplastic in one and squamous in one). Eleven patients had grade 1 disease (33.0%) while eight patients (24.2%) had grade 2 and fourteen patients (42.4%) had grade 3 disease. Maximal tumor diameter was <10 cm in eighteen patients (54.5%) and ≥10 cm in the remaining fifteen patients (45.5%). Eighteen patients had right ovarian tumor and remaining 15 had left sided tumors. Clinicopathological features of patients are shown in Table 1.

**Table 1:** Clinico-Pathological Features of patients

Features of Patients	Number of patients	%
**Age**		
< 50	11	33.3
≥50	22	66.7
**Gravida**		
0	3	9.1
1-3	14	42.4
≥4	16	48.5
**Parity**		
0	3	9.1
1-3	23	69.7
≥4	7	21.2
**Pre-operative Ca-125**		
≤ 35	12	37.5
35-500	17	50
≥500	4	12.5
**Menopausal status**		
Premenopausal	21	63.6
Postmenopausal	12	36.4
**Histology**		
Serous	7	21.2
Musinous	8	24.2
Endometrioid	5	15.2
Other*	13	39.4
**Grade**		
1	11	33.3
2	8	24.2
3	14	42.4
**Maximal Tumor Diameter**		
< 10 cm	18	54.5
≥10 cm	15	45.5

Other*: Clear cell in four, mixed type in four, transitional in three, anaplastic in one and squamous in one patient

All the patients were assumed to have tumors in clinical stage Ia after pre-operative and intra-operative evaluations. However, twelve patients (36.2%) were found to have microscopic metastasis after post-operative pathological evaluations and therefore had upstaged diseases. Seven of these upstaged patients (21.2%) had capsular invasion and were upstaged to Ic. One patient (3.0%) had microscopic omental metastasis with ovarian capsular invasion and was upstaged to IIIa. Two patients (6.0%) had ipsilateral ovarian capsular invasion with contralateral ovarian involvement were upstaged to stage Ic. The remaining two upstaged patients (6.0%) had lymphatic metastasis and were classified in stage IIIc disease. Two patients with capsular invasions had serous histology (one grade 1 and one grade 3), two had mucinous carcinoma (grade 1), one had clear cell carcinoma (grade 3), one had squamous cell (grade 3) and one had mixed serous and mucinous carcinoma (grade 2). The patient with omental metastasis had mucinous carcinoma (grade 3). One patient with contralateral ovarian involvement too had mucinous carcinoma (grade 2) while other had serous carcinoma (grade 2). Para-aortic lymph nodes involvement was seen in one patient (left sided tumor) and ipsilateral pelvic lymph nodes in another. Therefore, it was decided that a unilateral pelvic lymphadenectomy would miss half the lymphatic metastasis. Each patient was found to have one (n = 1) metastatic lymph node. Tumor histology was serous and transitional cell in these two patients with lymphatic metastasis. All patients with lymphatic metastasis had grade 3 disease. None of these patients had a capsular invasion or involvement of the contralateral ovary or positive peritoneal cytology.

## Discussion

Lymphadenectomy is a routine part of surgical staging in epithelial ovarian carcinomas [8]. Despite the vast amount of data detailing the role and extent of lymphadenectomies in published literature, there are still many questions that need to be answered. These debates are particularly important for unilateral tumors apparently confined to the ovaries. Questions regarding lymphadenectomies include: the limits of lymphadenectomies for these tumors, the sufficiency of performing a unilateral pelvic lymphadenectomy, the need to perform a para-aortic lymphadenectomy for patients and the role of surgical staging in these patients. These questions are particularly important if one also considers the morbidity of lymphadenectomy and staging laparotomy. The state of patients with unilateral tumors with strict criteria as mentioned above (clinical stage Ia) is another debate; are systematic lymphadenectomies really necessary or in other words, is retroperitoneal metastasis really possible without any intra-abdominal metastasis? And which lymph nodes are the first to be metastasized?

There are a limited number of published reports evaluating the role of lymphadenectomies in early staged ovarian cancers [2,5-7,9-20]. All the studies are of a retrospective nature and usually had small sample sizes. From these studies, lymphatic metastasis is thought to be present in about 4-27% of early staged patients [2,6,10,12,15,16,21,22]. This large heterogenity in lymphatic metastatic rates is mainly related to the type of lymphadenectomy performed in these studies (sampling vs. systematic, only pelvic or isolated unilateral pelvic etc.). Our previous study reported a of 13% including clinical stage I-II patients [23]. Previous reports were also excessively heterogeneous with respect to analyzed variables such as substages, grades, tumor histology and extend of lymphadenectomies so that it was almost impossible to collect all of these studies under a single Table (Table 2 and 3). Majority of previous studies also included patients with greater than clinical stage Ia disease [2,7,10].

**Table 2:** Pelvic and paraaortic involvement in early staged patients with epithelial ovarian carcinoma

Author	Number of patients	Number of isolated Pelvic LN positive patients	Number of isolated aortic LN positive patients	Number of Pelvic and aortic positive patients
Petru et al. [2]	40	7	1	1
Benedetti Panici et al. [6]	37	3	2	0
Onda et al. [10]	59	3	2	8
Sakai et al. [12]	63	2	2	0
Kanazawa et al. [22]	68	5	5	4
Morrice et al [15]	100	3	13	7
Harter et al [16]	70	0	4	4

Total	437	23 (%5.2)	29 (%6.6)	24 (%5.5)

**Table 3:** Previous studies evaluating the contralateral pelvic and paraaortic lymphatic metastasis presenting in unilateral clinical stage I or stage Ia epithelial ovarian carcinoma patients (stage Ia patients are bolded and ipsilateral pelvic metastasis are not included.).

Author	Description of Study and Stages	Tumor Laterality	Reported Histology and Grade (Gr)	CPLN or PA Met*	n
Walter J. [5]	**Ia** (case report)	Left **(Ia)**	Anaplastic, Gr3	Right paraaortic + right pelvic	1

Onda [10]	7 out of 33 Stage I patients have lymphatic metastasis. 6 have unilateral tumor.	Left	Not defined	Bilateral pelvic+PA	1
		Right		Right PA	1
		Right		Right pelvic+Right PA	1
		Right		Left pelvic+Bilateral PA	1

Cass [10]	96 unilateral stage I patients with unilateral or bilateral pelvic paraortic lymphadenectomy are evaluated. 14 of these 96 had lymphatic metastasis.	Right	Gr3	Left PA	1
		Right	Gr3	Left pelvic	1
		Left	Gr3	Right PA +Right pelvic	1
		Left	Gr3	Bilateral pelvic	1
		Right	Gr3	Bilateral pelvic	1
		Right	Gr3	Right PA	4
		Left	Gr3	Left PA	2

Petru [2]	9 out of 40 Stage I patients had lymphatic metastasis. 6 out of 9 patients had unilateral tumors.	Not defined	Not defined	Contralateral pelvic	1
				Pelvic+paraaortic	1

Negishi[9]	8 out of 123 stage I patients had lymphatic metastasis. One patient was **stage Ia**.	Right **(Ia)**	Clear cell (Both)	Right paraaortic	2
		Left	Clear cell (Both)	Bilateral PA	2
		Left	Mucinous (grade ?)	Bilateral pelvic and PA	1
		Left	Clear	Left PA	1
		Left	Mucinous	Left PA	1
		Left	Serous	Left PA	1

Sakuragi [13]	4 out of 78 Stage I patients had lymphatic metastasis. One patient out of 31 **Stage Ia **had lymphatic metastasis.	Right (**Ia**)	Clear cell Gr1	Right PA	1
		Left	Clear/Mucinous Gr2	Left PA	2
		Left	Clear cell Gr1	Bilateral PA	1

Morrice [15]	8 out of 60 **stage Ia **patients had lymphatic metastasis.	Not defined	Gr2:4	Contralateral PA	1
		Not defined	Gr3:3	Ipsilateral PA	5
				Ipsilateral PA+Pelvic	1

Baiocchi, [20]	32 out of 242 Stage I patients had lymphatic metastasis. 24 out of 32 was in **Stage Ia**. Total number of stage Ia was 215.	Right	Overall distribution	Left pelvic	1
		Right	of grades among	Left PA	2
		Right	patients with	Bilateral pelvic+PA	3
		Right	lymphatic metastasis	Right PA	5
		Right	was as follows:	Right pelvic+PA	2
		Left	Borderline: 7	Left PA	4
			Gr1: 3		
			Gr2: 6		
			Gr3: 15		
			Undefined: 1		

Suzuki [14]	5 out of 47 Stage I patients had lymphatic metastasis. 18 patients had **Stage Ia **disease.	Not defined **Ia**	Clear cell Gr2	Contralateral pelvic	1
		Not defined	Serous Gr1	Contralateral pelvic	1
		Not defined	Serous Gr2	Ipsilateral pelvic+PA	1
		Not defined	Serous Gr2	PA	1
		Not defined	Serous Gr1	Ipsilateral suprainguinal	1

This Study	2 out of 33 stage Ia patients had lymphatic metastasis	Left	Transitional Gr3	PA	1

* Contalateral Pelvic or Paraaortic Lymph Node Metastasis in Stage I Epithelial Ovarian Carcinoma

From these studies, we can conclude that lymphatic metastasis ranges from around 4-27% of clinical stage I patients with epithelial ovarian carcinomas. Both pelvic and para-aortic lymph nodes may be involved in these early staged patients. The number of studies evaluating the laterality of lymphatic metastasis is even lower (Table 3) and contrary to our findings; those studies found that contralateral pelvic lymphatic metastasis could be seen in a significant percent of clinical stage I patients. This was also true for clinical stage Ia patients (Table 3). All previous literature point a high rate of contralateral pelvic metastasis and recommends a bilateral pelvic lymphadenectomy except for the study by Benedetti-Panici et al [6]. They evaluated 35 unilateral clinical stage I patients. Three patients had a metastasis on ipsilateral pelvic nodes, and two other patients had paraaortic metastasis and the authors recommended an ipsilateral lymph node dissection to be appropriate for staging and therapeutic purposes [6].

Tumor grades were not reported homogeneously in all reports. Among the reported cases, majority of the lymphatic metastasis was seen in high grade (grade 3) tumors or in unfavorable tumor histologies (transitional, clear cell etc.). However, there were also reports of low grade serous tumors with lymphatic metastasis (Table 3). In our report, also two patients with lymphatic metastasis had grade 3 disease and one patient had an unfavorable tumor histology (transitional cell). An accurate frozen analysis may direct the physicians to allocate patients for systematic lymphadenectomy.

This report is unique in that it is the first prospective study evaluating this topic. All the patients had undergone a full surgical staging with systematic bilateral pelvic and para-aortic lymphadenectomies. Our results were similar to previous reports, when one considers the overall rates of upstaging (36.2%), rates of lymphatic upstage (6%) and the localizations of lymphatic metastasis (pelvic and para-aortic regions were involved) [2,4,5,7,9-20]. And combined with the previous available data (Table 2 and 3), this study prospectively shows the necessity of a systematic lymphadenectomy including paraaortic region, even in clinical stage Ia low grade patients.

## Conclusion

In clinical stage Ia ovarian cancer patients, there may be a risk of paraaortic and pelvic lymph node metastasis. Further studies with larger sample size are needed for an exact conclusion. Currently, considering with the previous literature (Table 2, 3); a systematic bilateral pelvic and paraaortic lymphadenectomy should be the state of art for clinical stage Ia patients.

## Competing interests

The authors declare that they have no competing interests.

## Authors' contributions

GD, primary corresponding author, participated in the design and coordination of the study, worked in all steps of the manuscript, MG, worked in collecting the data and making statistical evaluations, AA and KY primarily responsible for the management of patients surgically and worked in critical revision and edition of manuscript, AU, evaluated pathological images. All authors read and approved the final manuscript.
